# Comparative cost-effectiveness analysis of the subacromial spacer for irreparable and massive rotator cuff tears

**DOI:** 10.1007/s00264-018-4065-x

**Published:** 2018-07-31

**Authors:** Alessandro Castagna, Raffaele Garofalo, Eran Maman, Alisha C. Gray, Elizabeth A. Brooks

**Affiliations:** 1 Humanitas Research Hospital, Via Manzoni 56, Rozzano, 20089 Milano, Italy; 2 Ospedale Miulli, Acquaviva delle Fonti, Bari, Italy; 3 Shoulder Unit, Orthopedic Surgery Division Tel Aviv Medical Center, Sackler Faculty of Medicine, Tel Aviv University, Tel Aviv, Israel; 4 Translational Technologies International, LLC, Hampstead, MD USA

**Keywords:** Biodegradable balloon, Subacromial spacer, Rotator cuff tear, Cost-effectiveness, Irreparable tears, Rotator cuff tear treatment modalities

## Abstract

**Purpose:**

There is ongoing debate regarding the optimal surgical treatment of irreparable rotator cuff tears (IRCT). This study aimed to assess within the Italian health care system the cost-effectiveness of subacromial spacer as a treatment modality for patients with IRCT.

**Methods:**

An expected-value decision analysis was created comparing costs and outcomes of patients undergoing arthroscopic subacromial spacer implantation, rotator cuff repair (RCR), total shoulder arthroplasty, and conservative treatment for IRCTs. A broad literature search provided input data to extrapolate and inform treatment success and failure rates, costs, and health utility states for these outcomes. The primary outcome assessed was an incremental cost-effectiveness ratio (ICER) of subacromial spacer implantation versus shoulder arthroplasty, RCR, and conservative treatment.

**Results:**

Subacromial spacer is favorable over both arthroscopic partial repair and shoulder arthroplasty since it costs less than both options and increases effectiveness by 0.06 and 0.10 quality-adjusted life years (QALYs), respectively. While conservative treatment is the least costly management strategy, subacromial spacer results in a gain of 0.05 QALYs for the additional cost of 522 €, resulting in an ICER of 10,440 €/QALY gain, which is below the standard willingness to pay ratio of $50,000 USD. Strategies with an ICER of less than 50,000 USD are considered to be cost-effective.

**Conclusions:**

Based on the available evidence and reasonably conservative assumptions, subacromial spacer is likely to provide a safe, effective, and cost-effective option for patients with massive IRCTs. Furthermore, this cost-effectiveness analysis may ultimately serve as a guide for development of health care system and insurer policy as well as clinical practice.

## Background

Rotator cuff tears (RCT) significantly influence the quality of life for patients and are one of the most common reasons for orthopaedic clinic visits. Approximately 10 to 40% of all RCTs are massive (greater than 5 cm in size or complete detachment of two or more tendons) [1–3]. Each year in the USA, approximately 4.5 million patient visits are due to shoulder pain, and the majority of them are associated with RC pathologies. A substantial proportion of these tears are determined to be irreparable on evaluation [4]. Thus, massive irreparable RCTs present a challenging clinical problem for both patients and orthopaedic surgeons.

Treatment modalities, such as physical therapy and arthroscopic debridement with biceps tenotomy, have been shown to reduce pain and improve quality of life [5, 6]. Nevertheless, surgical treatment options, such as hemiarthroplasty (HA) and reverse total shoulder arthroplasty (rTSA), may result in better functionality achievements [7, 8]. Recent studies have shown that RTSA has the potential to achieve better functional outcomes compared with other treatment strategies [9]. However, RTSA has been associated with higher complication and re-operation rates as well as greater costs [8, 10].

The InSpace™ technology system, commonly known as “balloon arthroplasty,” is a more recent development for massive RCTs [11]. InSpace™ offers a minimally invasive surgical technique using a biodegradable subacromonial balloon-shaped spacer implanted between the humeral head and acromion that enables frictionless gliding to restore shoulder biomechanics.

**Fig. 1:**
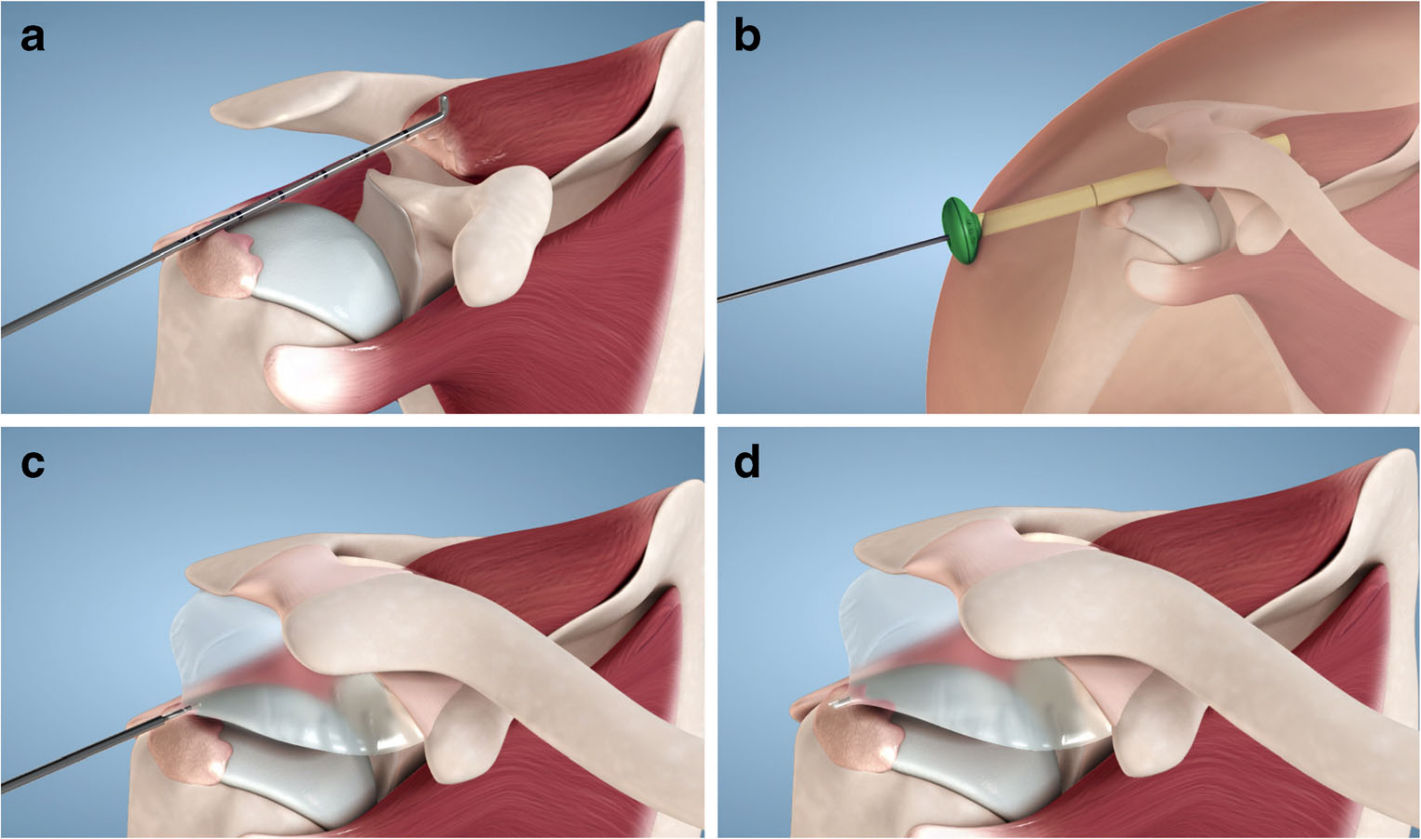
Schematic presentation of the InSpace™ system implantation steps: **a** Step 1: selection of the correct spacer size by measuring the distance from the lateral border of the greater tuberosity to approximately 1–2 cm medial to the glenoid apex, using a surgical probe. The system is available in three optional sizes (small, medium, or large) to accommodate individual patient’s anatomic variations. **b** Step 2: insertion of the InSpace™ introducer into the subacromial space (the spacer is folded inside). **c** Step 3: inflation of the balloon-shaped spacer with saline as per spacer size and device’s instruction for use. **d** Step 4: final position of the sealed spacer in the subacromial space between the humeral head and acromion

The InSpace™ device can be safely and quickly inserted using either standard arthroscopic procedure or as reported by some authors using fluoroscopic guidance (Fig. 1). Such fluoroscopy-guided insertion is done under local anaesthesia on an outpatient basis [12].

With shorter rehabilitation, this less invasive technique may be a viable surgical alternative for patients who wish to delay or have contraindications to more invasive surgical procedures.

The data regarding the cost-effectiveness of treatment options for massive irreparable RCTs is scarce. Recent study by Longo et al. confirmed that the socioeconomic burden of RC surgery is growing and heavily affecting the working population in Italy [13]. The goal of this study is to evaluate the cost-effectiveness of InSpace™ technology as an alternative for initial treatment of irreparable rotator cuff tears (IRCT) within the health care system of Italy. The primary aims of this study were as follows: (1) to determine the relative cost-effectiveness of four common treatment strategies for massive irreparable RCTs using a decision analytic model and (2) to perform sensitivity analyses of the probabilities, utilities, and costs inputs to account for model accuracy and precision.

## Methods

This economic evaluation uses an expected-value decision analytic model to compare estimated 24-month outcomes associated with the management of IRCT in terms of cost per quality-adjusted life years (QALYs) gained. The following treatment alternatives were considered: (1) InSpace™, (2) reverse total shoulder arthroplasty (rTSA), (3) partial repair/debridement, or (4) non-operative care. A comprehensive literature review was conducted to guide values of the key model effectiveness parameters. Base inputs and ranges for parameters were obtained from the literature based on the following hierarchy: (1) quantitative synthesis of multiple high-quality research papers, (2) findings from other cost-effectiveness studies, (3) evidence from specific individual high-quality studies, and (4) expert opinion.

### Model structure and computation

**Fig. 2:**
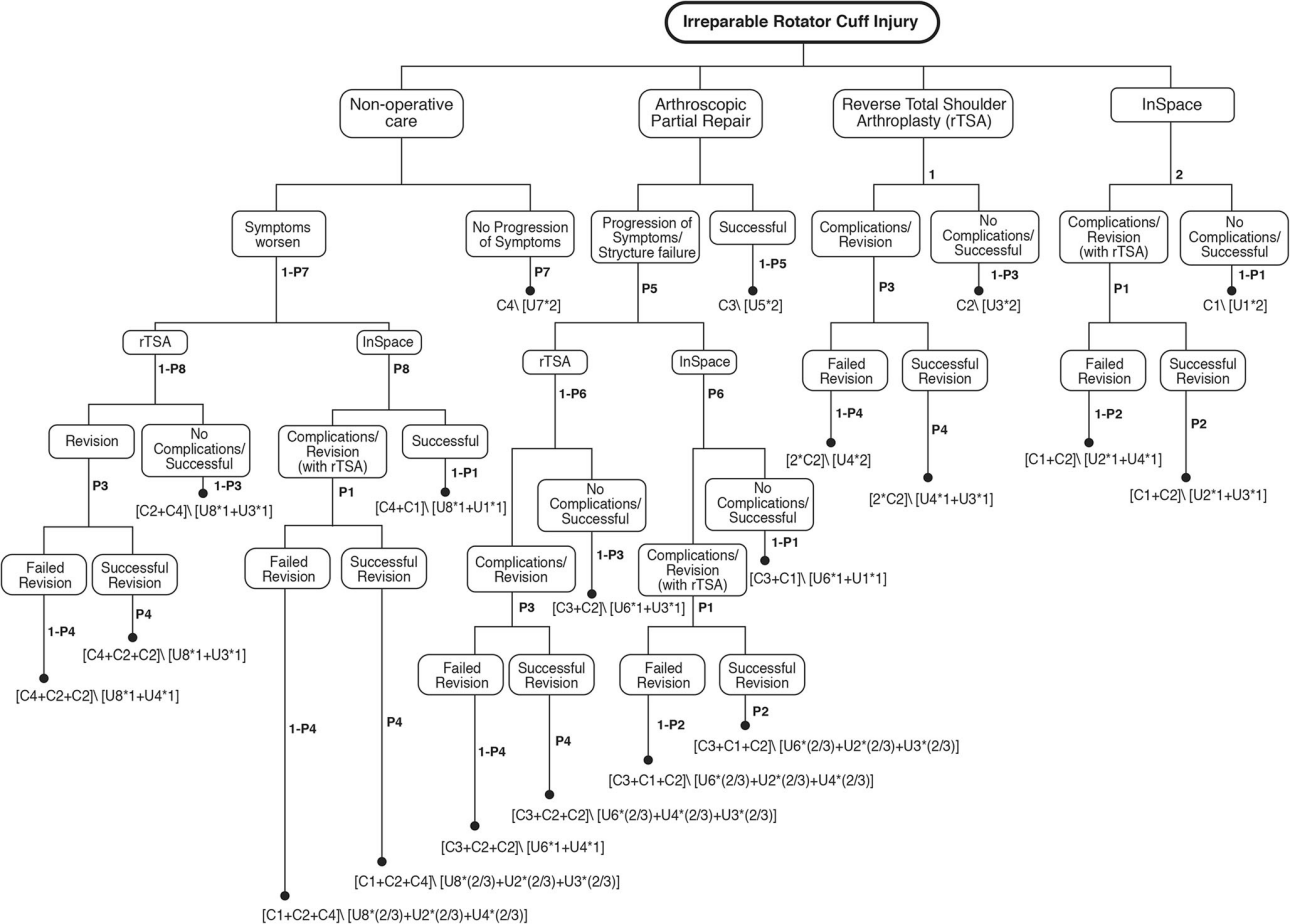
Model structure—treatment alternatives for irreparable rotator cuff tear

The model was built and analyzed with the software TreeAge Pro Healthcare 2017. For each IRCT treatment strategy, the course of treatment and outcomes are represented (Fig. 2). A probabilistic subset of those patients will have success with a treatment or course of action, while the remaining will require revision or another treatment, which also has a probability of success or failure, based on the prior treatment and outcome. As shown in Fig. 2, if a patient begins with non-operative care but symptoms worsen, they will proceed to either rTSA or InSpace™. For patients undergoing partial repair, they may either have a successful procedure or require a revision. As with patients receiving non-operative care, if they require revision, they will either undergo rTSA or receive InSpace™. For patients initially undergoing rTSA, their procedure will either be successful or will require a revision procedure. If they require a revision, the revision will either be successful or fail. If the revision fails, they will remain in that health state. If a patient initially utilizes InSpace™, they will either have no complications or require a revision (rTSA) which will either be successful or fail. If the revision fails, the patient will remain in that health state. Thus, a weighted average of the possible returns for each alternative (costs and QALYs), with probabilities used as weights, is calculated.

### Model assumptions and parameters

In the model, we operated under several assumptions regarding the patient treatments and costs: (1) all patients entering the model are diagnosed with IRCT; (2) prior to entering the respective treatment arms, patients underwent equivalent prior non-operative care of 6 weeks with equivalent costs; (3) costs included direct medical care costs; (4) post-surgery rehabilitation costs were not included due to a lack of studies containing that information; and (5) patients who experience complications following surgical care require revision surgery. Additionally, model parameters, displayed with their corresponding data sources in Table 1, consist of costs, health utilities, and transition probabilities. Costs represent direct medical costs to the health care system and were sourced from published literature and based on US Medicare cost of surgical costs, including the cost of the procedure, implant costs, and costs of hospital care. All costs were normalized to 2017 US dollars using a cumulative inflation rate based on the consumer price index (CPI) for health care services (use Sundar source). They were then translated into Italian health care Euros using the purchasing power parity (PPP) conversion factor, which is the number of units of the country’s currency required to buy the same amounts of goods and services in the domestic market as the US dollar would buy in the USA, currently 0.75 for Italy. PPPs show the ratio of prices in national currencies of the same good or service in different countries. They were then translated into Italian health care system Euros using a purchasing power parity (PPP) of 0.75. The cost of InSpace is assumed to be less than the cost of rTSA and similar in cost to arthroscopic repair. This input was validated by a review of Italian hospital charges. As reduced quality of life (QoL) is associated with both disease treatment and disease health outcomes (Whitehead 2010), we incorporated QoL for each health outcome of interest using estimates of utilities reported in the peer-reviewed literature. In this analysis, health utilities measure quality of life on a 0–1 scale anchored by death (0) and perfect health (1) and are associated with each IRCT treatment outcome. The primary patient outcome of interest for this analysis is the total quality-adjusted life years (QALYs) experienced by the patient over the course of a 24-month period. The timeframe was chosen based on the length of follow-up time in recently published studies. We assume that treatment decisions (i.e., revision) are made within one year, allowing a year of follow-up time. Utilities are used to calculate quality-adjusted life years (QALYs) experienced by a patient by decrementing the amount of time that has passed by the health utility experienced during that time period. For example, a patient experiencing perfect health (i.e., health utility = 1) for a period of two years will experience 2 QALYs during that 2-year period. A patient experiencing a health utility of 0.5 will experience 2 × 0.5 = 1 QALY during that two year period.

**Table 1:** Model parameters with base case and sensitivity analysis range

	Description	Base case	Range from lit review	References
Costs (€)				
Variable				
C1	Cost InSpace™	15,000	12,000–18,000	Assumption based on Makhni and Kang 2016
C2	Cost of total reverse shoulder arthroplasty	28,210	17,619–55,359	Renfree 2013, Virani 2013, Makhni 2016, Kang 2016
C3	Cost of partial arthroscopic repair	11,739	8306–17,413	Makhni 2016, Vitale 2007, Genuario 2012, Mather 2013, Bisson, 2015, Kang 2016
C4	Cost non-operative care	9068	7254–10,882	Makhni 2016
Utilities				
U1	Successful InSpace™	0.700	0.560–0.840	Assumptions
U2	Complication InSpace™	0.660	0.520–0.792	Assumptions
U3	Successful rTSA	0.660	0.600–0.900	Makhni 2016, Kang 2016
U4	Complication rTSA	0.411	0.411–0.700	Makhni 2016, Kang 2016
U5	Healed partial repair	0.662	0.510–0.770	Makhni 2016, Kang 2016
U6	Re-torn partial repair	0.656	0.470–0.710	Makhni 2016, Kang 2016
U7	No progression of symptoms non-operative care	0.662	0.56–0.84	Makhni 2016, Kang 2016
U8	Symptoms worsen (retear) Non-operative care	0.660	0.56–0.84	Makhni 2016, Kang 2016
P1	Probability of revision (InSpace™)	0.125	0.40–0.125	Gervasi 2016, Study PowerPoint
P2	Probability of successful revision (InSpace™)	0.50	0.40–0.60	Assumption, Makhni 2016
P3	Probability of revision (rTSA)	0.10	0.10–0.69	Anley 2014, Russo 2015, Kang 2016,
P4	Probability of successful revision (rTSA)	0.50	0.10–0.84	Anley 2014, Russo, Makhni 2016 2015, Kang 2016, Holschen 2017
P5	Probability of structure failure with arthroscopic partial repair	0.52	0.10–0.52	Berth 2010, Kang 2015
P6	Probability of InSpace™ if arthroscopic partial repair structure fails	0.50	0.40–0.60	Assumption
P7	Probability of success with Non-operative care	0.68	0.68–0.82	Mather 2013, Kang 2016
P8	Probability of InSpace™ if symptoms persist with Non-operative care	0.50	0.40–0.60	Assumption, Makhni 2016

Movement through the decision tree, shown in Fig. 2, is governed by (1) the success or failure rates of the various IRCT management strategies; (2) the likelihood of revision procedures; and (3) the success or failure rates of the revision procedures. These model transition probabilities were derived from the literature and their values and data sources are also displayed in Table 1. As hypothetical patients traverse the model, applicable costs and outcomes (health utilities) associated with the treatment alternatives for IRCTs accrue. Using the accrued cost and accrued utilities as the effectiveness parameter for each potential path through the model, the model evaluates the expected cost and expected QALYs based on the transition probabilities for each treatment strategy.

### Cost-effectiveness analysis

All analyses were conducted by following the recommendations from the ISPOR Panel on Cost-effectiveness in Health and Medicine. Comparisons of strategies were made using the incremental cost-effectiveness ratio (ICER), which is the ratio between the difference in costs and QALYs of each strategy compared to the previous strategy, when strategies are ordered by cost, e.g., incremental dollars per QALY. A willingness to pay (WTP) threshold for the additional cost incurred for each change in QALYs is established. Typically, strategies with an ICER of less than 50,000 US dollars (45,483 €) are considered cost-effective. A strategy is dominated when another strategy is both less costly and more effective and is dominant when it costs less and is more effective than another alternative. A strategy with a negative ICER is dominated, reflecting the additional cost per loss in QALYs. Incremental analysis is critical for decisions regarding the allocation of scarce resources. The “base case” analysis is the scenario which involves evaluating the model with each parameter at its estimated value. One- and two-way sensitivity analyses were performed to explore the impact of varying key parameter values from the base case on the model results.

## Results

### Base case analysis

**Fig. 3:**
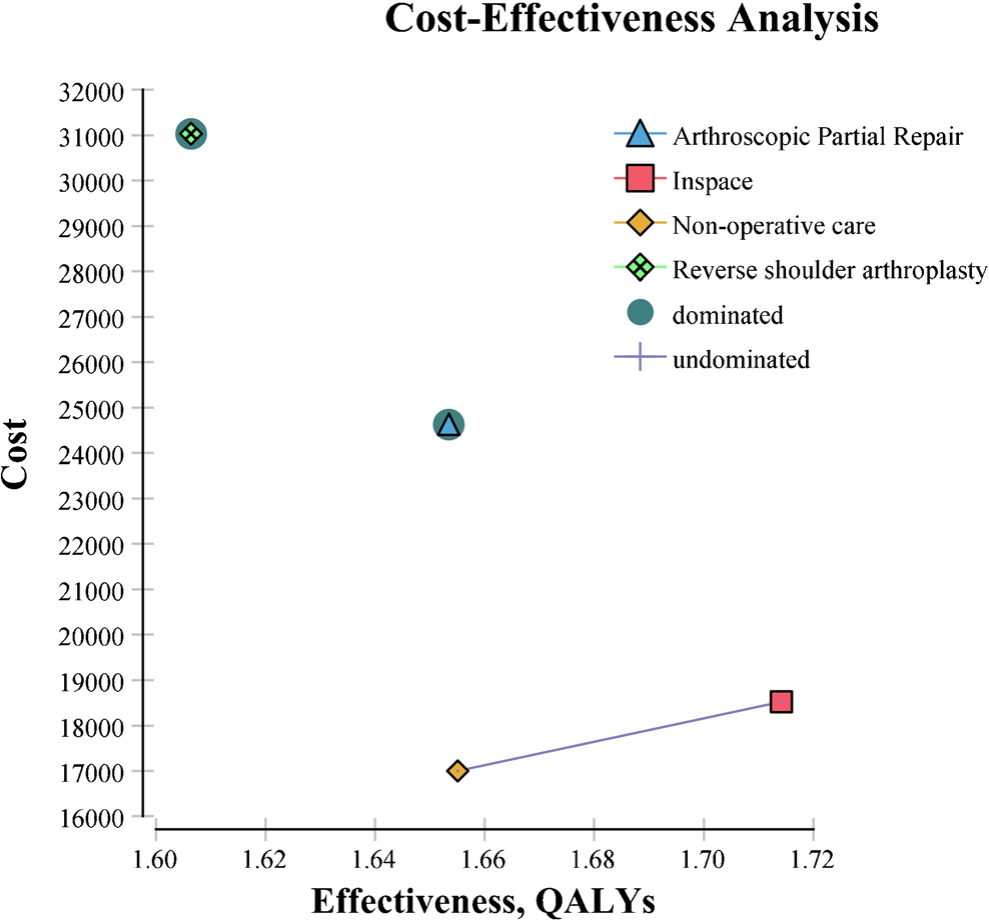
Graph of base case cost-effectiveness analysis. The four competing treatment strategies with model parameters set at the base case scenario (QALYs, quality-adjusted life years)

The expected costs, QALYs, and ICERs associated with each treatment alternative are presented in Table 2. After conducting the base case analysis, the study finds that InSpace™ is the dominant strategy, meaning that it is both less costly and more effective than all other treatment alternatives (Fig. 3). InSpace™ dominates both arthroscopic partial repair and reverse shoulder arthroplasty since it costs less than both options and increases effectiveness by 0.06 and 0.10 QALYs, respectively. While non-operative care is the least costly management strategy, InSpace™ results in a gain of 0.05 QALYs for the additional cost of 522 €, resulting in an ICER of 10,440 €/QALY gain, which is below the standard willingness to pay ratio of $50,000 US dollars.

**Table 2:** Base case cost-effectiveness analysis

Management strategy	Total expected per patient cost (€)	Total expected per patient QALYs	Incremental cost (€)	Incremental effectiveness QALYs	Incremental C/E (ICER)
Non-operative care	16,805	1.33			
InSpace™	17,327	1.38	522	0.050	10,440
Arthroscopic partial rotator cuff repair	24,312	1.32	6985	− 0.060	− 116,417
Reverse shoulder arthroplasty	31,031	1.28	6719	− 0.040	− 167,975

Arthroscopic partial repair and rTSA are both dominated since both are more costly and less effective than InSpace™ and non-operative care. Arthroscopic partial repair is more effective but slightly more costly than non-operative care. When a WTP of $50,000 (45,483 €) is used, InSpace™ is the preferred strategy. *rTSA* reverse total shoulder arthroplasty, *WTP* willingness to pay

Typically, strategies with an ICER of less than 50,000 US dollars (45,483 €) are considered cost-effective. Thus, InSpace™ is the most cost-effective strategy and may be a preferable treatment strategy for the management of irreparable rotator cuffs.

### One-way sensitivity analysis

A one-way sensitivity analysis was conducted on key variables including cost of InSpace™, probability of InSpace™ revision, health utility associated with InSpace™ success, probability of success of non-operative care, cost of reverse total shoulder arthroplasty, cost of arthroscopic partial repair, and cost of non-operative care. In the sensitivity analysis, the parameter range utilized was based on a range of values established in the literature, where applicable, or was allowed to vary (one at a time) up or down by 20%. Notably, even if the cost of InSpace™ is increased by 20% or the probability of success of the non-operative strategy decreases to 87.5%, it is not dominated by an alternative treatment option. Further, if the cost of InSpace™ decreases by 20%, the probability the success of InSpace™ increases to 96%, or if the cost of non-operative care increases to $17,327 US dollars, then InSpace™ dominates all strategies. However, if the utility associated with InSpace™ success drops by 20%, then non-operative care provides higher QALYs than InSpace™ at lower costs. The results of the one-way sensitivity analysis are shown in Table 3.

**Table 3:** One-way sensitivity analysis results

Alternative treatment options vs. InSpace™	Total expected per patient costs (€) for InSpace™	Total expected per patient QALYs for InSpace™	InSpace™ comparison
Cost of InSpace™, 12,000	14,327	1.38	InSpace™ costs lower, InSpace™ QALY higher InSpace™ dominates all strategies
Cost of InSpace™, 18,000	20,327	1.38	Non-op costs lower, InSpace™ QALY higher
Probability of InSpace™ success, 0.875	16,997	1.32	Non-op costs lower, InSpace™ QALY higher
Probability of InSpace™ success, 0.96	16,128	1.39	InSpace™ costs lower, InSpace™ QALY higher InSpace™ dominates all strategies
Probability of non-operative success, 0.54	17,327	1.38	InSpace™ costs lower, InSpace™ QALY higher InSpace™ dominates all strategies
Probability of non-operative success, 0.82	17,327	1.38	Non-op costs lower, InSpace™ QALY higher
Utility of InSpace™ success, 0.56	17,327	1.13	Non-op costs lower, non-op QALY higher InSpace™ dominated by non-op
Utility of InSpace™ success, 0.84	17,327	1.64	Non-op costs lower, InSpace™ QALY higher
Cost of rTSA, 17,619	17,327	1.38	Non-op costs lower, InSpace™ QALY higher
Cost of rTSA, 55,359	17,327	1.38	Non-op costs lower, InSpace™ QALY higher
Cost of arthroscopic partial repair, 8306	17,327	1.38	Non-op costs lower, InSpace™ QALY higher
Cost of arthroscopic partial repair, 17413	17,327	1.38	Non-op costs lower, InSpace™ QALY higher
Cost of non-operative care, 7254	17,327	1.38	Non-op costs lower, InSpace™ QALY higher
Cost of non-operative care, 10882	17,327	1.38	InSpace™ costs lower, InSpace™ QALY higher, InSpace™ dominates all strategies

*Non-op* non-operative, *rTSA* reverse total shoulder arthroplasty, *QALY* quality-adjusted life year

### Utility sensitivity analysis of surgical treatments

**Fig. 4:**
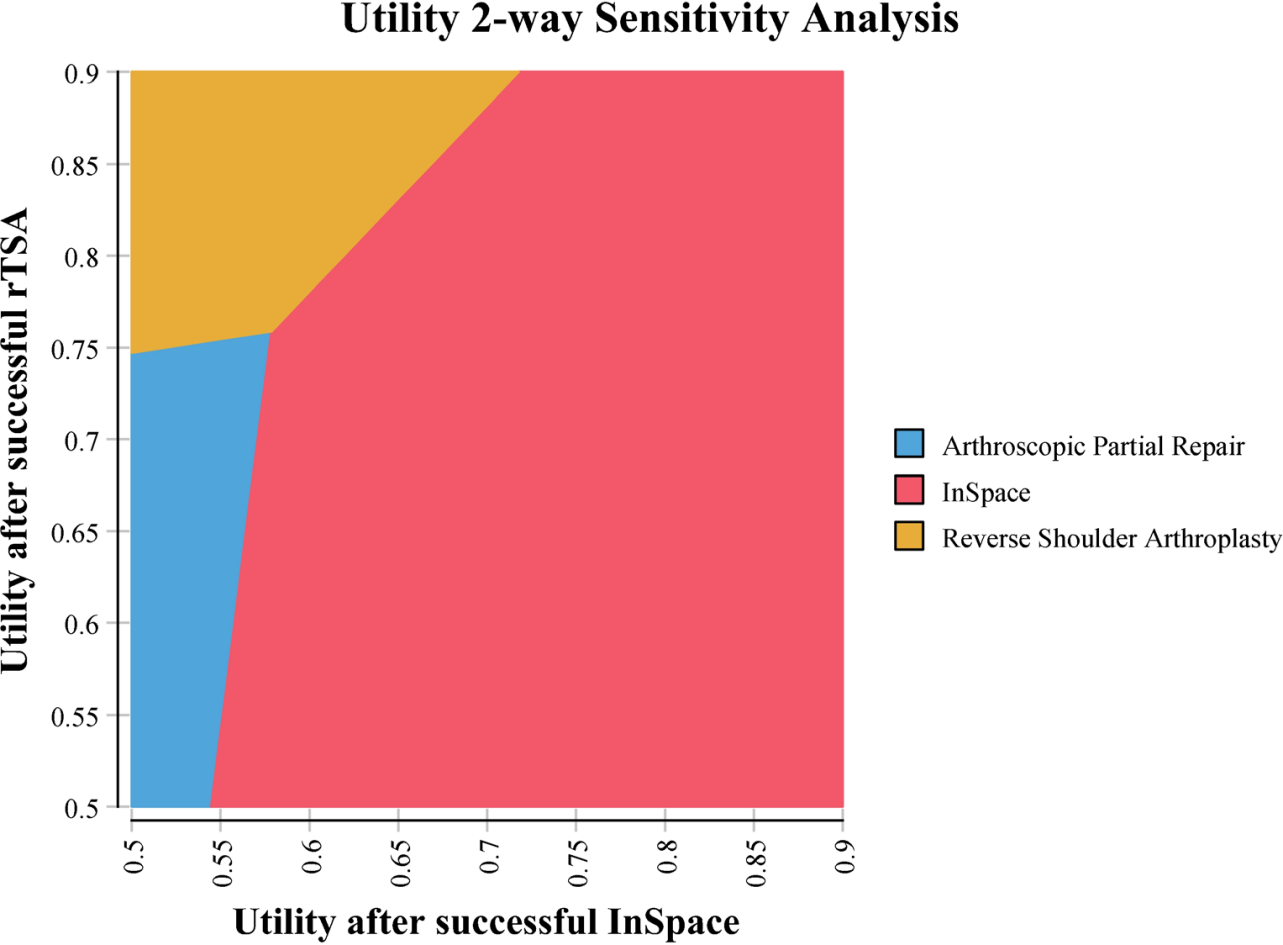
Two-way sensitivity analysis plot for utility after successful InSpace™ versus utility after successful rTSA. The colors represent the preferred strategy for the combination of the two parameters based on NMB when a WTP of $50,000 (45,483 €) is used. The InSpace™ strategy is the preferred strategy for most feasible combinations of InSpace™ utility and rTSA utility in a comparison of the three surgical treatments

Figure 4 shows the results of the two-way sensitivity analysis between the utility of successful InSpace™ and successful rTSA in a comparison of the three surgical treatments. Across the grid, the colours represent the preferred strategy based on the net monetary benefit (NMB) using a WTP strategy of $50,000 US dollars (45,483 €). InSpace™ was preferred with a utility greater than 0.55 and a difference no greater than 0.20 between it and rTSA, while rTSA was only preferred if the utility difference was larger than 0.20. Partial arthroscopic repair was only preferred when the utility of InSpace™ was below 0.55 and utility of rTSA below 0.75.

## Discussion

The principal results of this cost-effectiveness study clearly indicate that InSpace™ is the most beneficial, in terms of patients and economic outcomes, when compared to rTSA, partial repair/debridement, and conservative treatment bestowing to the existing body of literature.

There is an unmet need to determine the value of operative treatment for RCTs, with value determined by reductions in costs to society from rotator cuff repair. The most clinically and cost-effective strategies available to treat IRCTs in the countries we assessed were those that enhanced reduced sequelae, improved mobility, and decreased the need for revisions, through either a reduced number of visits or improved follow-up. It should be noted that while this analysis uses Italian perspective costs, the model could be analyzed utilizing cost data from other countries to develop additional cost-effectiveness analyses. One key finding of this analysis indicates that InSpace™ is the most cost-effective IRCT treatment strategy if the WTP threshold per QALY gained is above 10,000 €, well below the standard WTP threshold of $50,000 US Dollars (45,483 €). Moreover, in all scenarios considered, with the exception of a 20% decrease in the utility after successful treatment, InSpace™ either provides the greatest QALY benefit or dominates all other strategies.

In order to further test the validity and robustness of our cost-effectiveness analysis, we surveyed several different Italian hospitals to estimate the appropriate costs associated with each IRCT treatment modality in this analysis. Costs in this sub-analysis represent direct medical costs to the Italian health care system. Italian hospital costs in 2017 Euros included the cost of the procedure, implant costs, and costs of hospital care. Using costs as collected in this survey, InSpace™ became the dominant treatment option. As compared to non-operative care, arthroscopic partial repair, and total shoulder arthroplasty, InSpace™ was expected to reduce costs by 848 €, 3476 €, and 8357 €, respectively, while also improving patients’ quality of life. However, it was determined that non-operative care is the dominant strategy if the failure rate of InSpace™ increased to 0.4. Similarly, non-operative care would be the preferred strategy, assuming a willingness to pay of $50,000 US dollars (45,483 €), if the health utility associated with InSpace™ success was reduced to 0.595. In sum, our key finding of InSpace™ as the most cost-effective strategy is quite robust, with non-operative care becoming the preferred strategy under only two circumstances (i.e., increased effectiveness of non-operative care and reduced health utility associated with InSpace™ success).

Although several studies [14, 15] have been conducted to evaluate the cost-effectiveness analysis of various treatments for IRCTs, including rTSA, partial repair, and non-operative care, to our knowledge, this is the first evaluation of InSpace™, an emerging technology. Recent publications [16, 17] have presented positive efficacy and safety outcomes with InSpace™ over varying periods of time, and coupling with our cost-effectiveness analysis also adds to the knowledge base regarding the benefits of this IRCT treatment compared to other alternatives.

Although our cost-effectiveness analysis is adaptable for various cost perspectives and patient populations and includes current IRCT treatment options and standards of care with published literature justification, this analysis is not without limitations. While model parameters were derived as much as possible from published articles, some of our analysis is based on assumptions due to a lack of data comparing InSpace™ to the other available treatment alternatives. In particular, the success and failure rate utilities of InSpace™ were assumed to be similar to that of partial repair surgery since it is also less costly and less invasive than rTSA. Similarly, while most costs were based on a summary of the literature, the cost of InSpace™ was estimated in relation to the cost of rTSA and from expert opinion of Italian health care costs. These costs, however, were verified with hospital charges as satisfying the range of costs applied in this analysis. To rectify these challenges, a range of ± 20% cost and utilities were assessed in the sensitivity analysis with results showing InSpace™ to be optimal in most conditions.

In this analysis, a 24-month time frame is employed. However, this was chosen to represent the common course of treatment and allows for model flexibility of multiple age groups. It also reflects the use of InSpace™ in a clinical setting as technology employed to delay in the short-term more invasive and costly surgery, such as rTSA. A recently published study showed the safety and efficacy of InSpace™ for five years, well beyond a 24-month period [17].

Although, this analysis is not specifically designed for a particular age group, the model has the flexibility to vary key parameters, in particular success and failure rate utilities. Future work may be required to explore the viability of InSpace™ for specific age groups and clinical outcomes across longer follow-up periods for IRCT treatment.

By necessity, the cost analysis reported in this study reflects the medical economics in Italy, which may be different in other countries. However, the study does provide incremental changes in a large variety of the patient’s metrics, which should provide surgeons in other countries with the tools to adjust this analysis according to their local situation. Recent study by Longo et al. [13] reported that rate of RC repair in Italian hospitals is increased between 2001 and 2014, confirming that the socioeconomic burden of RC surgery is mounting. Approximately 65% of RC repairs were performed annually in patients younger than 65 years, thus heavily affecting the working population. According to the prediction model, hospital costs sustained by the national health care system for RC procedures are expected to be over one billion € by 2025 [13]. Current analysis does show that InSpace™ is cost-effective even in the high-cost environment of Italy, and we assume it would also then be cost-effective in many other counties within EU and the rest of the world.

Based on the available evidence and reasonably conservative assumptions, InSpace™ is likely to provide a safe, effective, and cost-effective option for patients with massive IRCTs. Furthermore, this cost-effectiveness analysis may ultimately serve as a guide for the development of health care system and insurer policy as well as clinical practice.
